# A Funny Symptom, Sulphur 55m

**Published:** 1890-09

**Authors:** M. A. A. Wolff

**Affiliations:** Gainesville, Tex.


### A FUNNY SYMPTOM, SULPHUR 55M.

M. A. A. Wolff, M. D., Gainesville, Tex.

At present an invalid in Pius Hospital, St. Louis, Mo. I am sixty-two years old. On May 19th, Professor Dr. Parsons, renowned for his skill as a surgeon, performed lithotomy (low) on my person. After about five weeks the wound was well healed and I was permitted to sit up. As long as the doctor visited me I followed strictly his orders, only occasionally making an extra prescription on my own hook, but with his approval.

On July 7th, he left for a summer vacation and appointed a young practitioner his amanuensis. I was then, and am yet under the impression that there were two symptoms he especially ordered him to watch for, the one the immense dropsy of the lower limbs, the other a bulging out of the abdomen on the left side about opposite the wound of incision, and a hardness under the swelling, in my opinion as of a scar, but said to be a steatoma. But besides these two there were plenty other symptoms which I shall enumerate as far as I can remember them. Recto-vesical fistula. Diarrhœa four to five times in twenty-four hours, of a fearful odor, and driven partly through the urethra. A protrusion from anus which I then took for prolapsus recti, but which the young doctor declares to be a pile, giving me great trouble in sitting down and after having been sitting comfortably even more unpleasantness when getting up. Incontinence of urine, which, however, only dribbles away. On and off a clogging-up sensation in urethra, deep, by mucus. The sediment is thick and stringy and even often comes out in stringy pieces from the urethra. Abundance of snow-white phosphates, once in a while even a little calculus, which has not been dissolved in the bladder, but which crumbles to flour between the fingers. A steady unpleasant coldness of scrotum. Many a time frothy discharge from urethra, caused by gas passing through the fistula. Very much flatulency ; on and after passing of blood coagula and examining the sediment find it blood-streaked. Much gurgling in abdomen as of water. Lastly, the funny symptom. When lying down always on back to sleep, I might just doze a little when I at once waken up make flatus sounds with the lips, splatter spittle and the tongue moves with rapidity in all directions.

To give an idea of its effect on other people, I shall state that one evening late, when I was under its influence, two of the sisters came scared to my room to find out what was the matter, and tell me that it disturbed the other patients on the floor. I can only compare it with the St. Vitus' dance of the oral and lingual muscles, with this difference, however, that the sufferer from St. Vitus' dance cannot control himself whilst in my own case opening the mouth wide and inhaling plenty of air stops the symptoms. Now the young doctor visited me every second or third day and prescribed, I understand, for the most prominent symptom, one time ordering me to take the medicine, always drops in water, every hour, next time every two hours and one every three hours. I kept a very exact sick record, which I gave him to read every time, hoping he would look out for the totality of symptoms. Thus time passed on with no, or hardly any change until July 18th, when Dr. W. L. Reed made a friendly call and kindly brought repertories and materia medicas to enable me to study my case up myself. However, after having talked it over he came to the conclusion that the simillimum would be Sulphur, and he dropped a few pellets on my tongue of the 55 M. From that moment the most astonishing change commenced. The urine got clear. I was able to pass it in a good stream, the feces natural, and thus not driven into the urethra, the dropsy diminished ; the funny symptom disappeared ; so that my only distress now is the pile, the fistula became improved and now the dropsy looks as if it would be all gone in a very few days. From the 18th to the 28th no other medicine was taken. On the 27th some fever and unpleasantness, which, however, was better after a hot bath, which seemed to open the urinary track. The doctor thought we might be warranted in taking another dose. This time the CM was given. This was yesterday, and I am under the impression that it has done me good. My study of the case, which lasted me three days (certainly only part of the day taken up for that purpose), gave the issue. Of about one hundred and twenty medicines indicated for several symptoms, Sulphur had the largest, viz. : twenty-one, of which five were in capitals, two in italics, not one of the others reaching the numbers twenty-one or five. I had all the time been lying on the back, now I can lie just as comfortable on either side. Thus far to date, should anything remarkable supervene you shall receive notice.

P. S.—I forgot to mention that urine at times comes gushing from anus, and—and that is important—that the bulging toward left groin is gone and to my feeling, the hardness greatly diminished (since I took the Sulphur).

